# Improving Industrial Quality Control: A Transfer Learning Approach to Surface Defect Detection

**DOI:** 10.3390/s25020527

**Published:** 2025-01-17

**Authors:** Ângela Semitela, Miguel Pereira, António Completo, Nuno Lau, José P. Santos

**Affiliations:** 1 Centre of Mechanical Technology and Automation (TEMA), Department of Mechanical Engineering, University of Aveiro, 3810-193 Aveiro, Portugaljps@ua.pt (J.P.S.); 2 Intelligent Systems Associate Laboratory (LASI), 4800-058 Guimarães, Portugal; nunolau@ua.pt; 3 Institute of Electronics and Informatics Engineering of Aveiro (IEETA), Department of Electronics, Telecommunications and Informatics, University of Aveiro, 3810-193 Aveiro, Portugal

**Keywords:** automated quality control, illumination, transfer learning, defect detection and classification, ResNet-50

## Abstract

To automate the quality control of painted surfaces of heating devices, an automatic defect detection and classification system was developed by combining deflectometry and bright light-based illumination on the image acquisition, deep learning models for the classification of non-defective (OK) and defective (NOK) surfaces that fused dual-modal information at the decision level, and an online network for information dispatching and visualization. Three decision-making algorithms were tested for implementation: a new model built and trained from scratch and transfer learning of pre-trained networks (ResNet-50 and Inception V3). The results revealed that the two illumination modes employed widened the type of defects that could be identified with this system, while maintaining its lower computational complexity by performing multi-modal fusion at the decision level. Furthermore, the pre-trained networks achieved higher accuracies on defect classification compared to the self-built network, with ResNet-50 displaying higher accuracy. The inspection system consistently obtained fast and accurate surface classifications because it imposed OK classification on models trained with images from both illumination modes. The obtained surface information was then successfully sent to a server to be forwarded to a graphical user interface for visualization. The developed system showed considerable robustness, demonstrating its potential as an efficient tool for industrial quality control.

## 1. Introduction

The technological advances in the industrial sector have significantly boosted the automation of the manufacturing process [1]. However, quality control still remains highly dependent on human intervention, whose subjectivity and inconsistencies might not guarantee the same quality standard for each product [2]. Painted surfaces, for instance, are quite prone to the occurrence of defects, that usually increase substantially in size as additional paint layers are applied. Previous reports have shown that human inspection failed to detect around 80% of minor defects on the painted surfaces of car parts [3]. This faulty quality monitoring can impact both customer satisfaction and production costs [4,5]. Hence, replacing this manual inspection for an automated detection system able to detect these surface defects would not only improve the monitoring’s accuracy and efficiency, but also increase the overall industry’s profitability [5].

Vision-based inspection systems in combination with deep learning (DL) algorithms have proven to be highly effective in the automotive industrial sector for defect detection on the surfaces of car bodies [2,3,4,6]. Given the reflective nature of these surfaces, most vision-based inspection systems employ deflectometry-based illumination for defect detection [2,4,7]. Despite its potential, the use of a single illumination strategy might not be sufficient for the detection of all surface defects, so the combination/fusion of the information retrieved from images with multiple illumination techniques has been reported with tremendous success [8,9]. Once this information is acquired and processed, it is fed to DL algorithms, such as convolutional neural networks (CNNs), for feature extraction and decision-making regarding defect detection and classification. In this instance, the classification process can be performed by either using a CNN built and trained from scratch, or by employing transfer leaning (TL) of a pre-trained CNN on large standard datasets [10,11]. While the self-built CNNs might enjoy more unique architectures and provide a better understanding of the data given the more accurate dataset, training a new network is time-consuming and it requires large datasets and constant parameter optimization [10]. On the other hand, transfer learning (TL) of a pre-trained CNN requires minimal efforts in building, training, and implementing the network, while allowing for the customization of the top layers for an improved performance in small datasets [12,13]. Since many CNN architectures can be subjected to pre-training for surface defect detection applications, the selection of the most suitable CNN should be based on their accuracy, required computational resources, and learning speed [14].

Heating devices, such as water heaters (electric and gas), boilers, and heat pumps, usually require a paint coating for aesthetic and protection purposes. Despite the extensive vision-based deep learning reports for surface defect detection, to the best of the authors’ knowledge, only a few reports applied this technology to the heating products [15]. The components of these products usually have different topographical features (specular and diffuse) that require a detection system with different illumination modes to effectively identify all possible defects on their surface. Hence, in the present work, an automatic vision inspection system was proposed for the detection and classification of defects on the painted surfaces of heating devices, which included the following: (1) an image acquisition module for dataset generation employing two illumination strategies (bright field and deflectometry-based lighting), (2) DL models for defect detection and classification with decision-level fusion, (3) painted surface information dispatching to a designed server, and (4) image graphical display. Regarding the feature extraction and decision-making performed by the DL models, two approaches were compared: (1) a CNN built and trained from scratch; and (2) TL of two pre-trained CNN architectures—ResNet-50 and Inception V3. At this point, the proposed system will not only provide a more enriched dataset in terms of variety given the wider detectable defect categories, but also an improved prediction accuracy regarding the status of the painted surfaces.

## 2. Related Work

Most industrial vision-based inspection systems comprise the following modules: (1) image acquisition, where the information pertaining to the surface is obtained in form of digital images using cameras or other sensors; (2) image processing, where the captured images are processed in order to highlight the defect’s features; (3) feature extraction, where specific features (such as color, texture, edges, among others) are extracted from the processed images; and (4) decision-making, where image classification is carried out using the extracted features to assess the status of the surface (defective or non-defective) [15,16]. Given their importance, the following subsections will focus on the illumination strategies for the image acquisition module, and the application of TL in the module of feature extraction and decision-making.

### 2.1. Illumination Strategies for the Image Acquisition Module

Several illumination approaches have been implemented in industrial vision-based inspection systems in order to enhance the visibility of the defects on the product’s surfaces, including bright field, dark field, and structured illumination [17]. Their selection is highly dependent on the surfaces’ features such as topography (flat or curved), roughness (diffuse or specular), and optical properties (reflection, transmission, or absorption) [18]. Bright field, for instance, is suitable for surfaces containing diffuse defects due to texture imperfections, as it allows for the generation of easily detected high-contrast regions. On the other hand, for specular surfaces, dark field and deflectometry-based structured illumination has significantly better performance in the presence of defects with three-dimensional features, given the significant deviations that occur from the original pattern [2,4].

Each of these illumination strategies are suitable for the detection of a specific class of defects, and therefore are unable to identify the entire repertoire of defects that can occur on these surfaces. In this instance, the combination of multi-modal fusion methods in a single inspection system has been explored to leverage complementary information from different modalities and allow for the identification of a wider range of defects [8]. These methods can be classified in three categories depending on the stage of the process in which fusion occurs: data-level, feature-level, and decision-level fusion [9,19].

The data-level fusion combines the images obtained from various illumination strategies in a single image before being fed to the module of feature extraction and decision-making [9]. Employing data-level fusion, Peng et al. developed a defect detection system for powder bed fusion processes using a multi-source acquisition module combining bright field and infrared imaging and a multi-sensor image fusion method. Their results revealed that this system effectively improved the image contrast and information richness, enhanced the display of image edge and texture information, and was able to retain and fuse the main information in the source image [20]. While promising, data-level fusion has some drawbacks that hinder its use for real-time application: (1) it provides redundant information; (2) it is prone to noise interferences; and (3) it has high computational cost [21].

The feature-level fusion, on the other hand, performs the extraction of features independently for each illumination strategy-based image to be later fused before the decision-making task [9]. Guan et al. also employed this feature-level fusion approach to create a multi-modal defect detection and classification method combining the deflectometry-based system and CNN, using the information from two different modalities: the light intensity contrast map and the original fringe pattern. In this instance, instead of using an alternate bright field source, the light intensity contrast map was calculated from the captured fringe images. The proposed multi-modal fusion network outperformed methods using either modality individually, achieving high classification accuracy (97%) for almost all defect categories (dirt, fingerprints, hairs and fibers, scratches, scuffing, and concave and convex defects) [7]. Even though the application of feature-level fusion significantly reduces the computational cost compared to data-level fusion, there is however the possibility of loss of information due to the fusion.

Conversely, the decision-level fusion employs two different decision-making models for each acquisition source for defect detection, whose outputs are then combined [9]. In this instance, besides being compatible with feature extraction of the information obtained from different illumination strategies/sources, this fusion method has low computational complexity and is suitable for real-time applications [21]. Using this approach, Hu et al. proposed an object detection system combining the output of two models classifying information retrieved from bright field and infrared imaging. The results indicated that this system effectively fuses the information from multiple decision-making models to improve the overall performance of the object detection [21]. Similarly, Cong et al. also employed a decision-level fusion method of bright field and infrared-based light that exhibited higher precision and robustness for object detection than single-modal methods [22]. Despite its more prevalent use with infrared imaging, this type of information fusion can be easily employed for other illumination strategies. Addressing this gap, this work will explore decision-level fusion in images retrieved from bright field and deflectometry-based structured illumination.

### 2.2. Transfer Learning for Feature Extraction and Decision-Making

CNNs have become one of the most prominent DL architectures for image classification and object detection, given their ability to automatically extract low-level features from the captured images into multiple layers and use them in hierarchical convolution to build high-level features later used for pattern analysis and classification [10,11,23,24]. CNN’s typical structure includes: (1) convolutional layers (CLs), which perform feature extraction to generate feature maps; (2) activation functions (AFs), such as rectified linear units (ReLu), to introduce non-linearity; (3) pooling layers (PLs), responsible for down-sampling the feature maps to reduce their computational complexity; and (4) fully connected layers (FCLs), which act as classifiers, providing probabilistic-based classification of the images [23,25,26,27].

Despite their potential, these networks require sizable training datasets in order to effectively learn how to recognize patterns and, ultimately, improve their performance [28]. Unfortunately, because of the low defect frequency observed in industrial inspections, obtaining a large and balanced dataset is quite unrealistic [29]. To properly address this issue, TL has been suggested given its compatibility with smaller datasets. The concept of TL is based on pre-trained models, which are trained in large public datasets, such as ImageNet, where they acquire transferable low-level features that are suitable to any image recognition task, regardless of its application [30]. Additionally, the pre-trained models are also customizable to improve their performance for the new task, either by simply modifying the top classification layers, or by performing fine-tuning, where the weights of the pre-trained model are continuously modified during the training for the new task [31]. The key advantages of TL include: (1) its smaller required labeled dataset; (2) its faster training process; (3) its lower required computational resources; and (4) its improved generalization attributed to extensive knowledge attained during training [32].

Since there is an extensive repertoire of available CNN architectures that have been subjected to pre-training [10,11,12], several factors should be considered for the selection of the more appropriate network, including their classification rate and accuracy, their required computational resources and their performance time [14]. In this instance, deeper networks, such as ResNet-152 and DenseNet-201, have outstanding performance in terms of classification accuracies, but exhibit longer performance time and require extensive computational resources. In comparison, lightweight networks, such as MobileNet, are well known for their reduced computational complexity and faster performance time to achieve average accuracies [33,34,35]. In this regard, several authors have performed performance comparisons on several pre-trained models. Berwo et al. compared several fine-tuned pre-trained CNNs for crack inspection in automotive engine components, namely AlexNet, Inception V3, and MobileNet. The results indicated that both Inception V3 and MobileNet, in combination with data augmentation techniques, achieved higher accuracy scores (100%), with MobileNet also requiring the lowest computational resources [10]. Singh et al. compared the performance of common pre-trained CNNs (VGG-19, GoogLeNet, ResNet-50, EfficientNet-b0, and DenseNet-201) for the identification of superficial defects in typical machined components, namely flat washers and tapered rollers. The results demonstrated the excellent performance of EfficientNet-b0 and ResNet-50 for defect detection, both achieving high accuracy scores in the lowest prediction time [12]. Zhang et al. also compared ResNet-50 with other pre-trained models (VGG19 and AlexNet) for defect detection, demonstrating its overall superior performance, reliability, and efficacy [36]. Conversely, the comparative analysis performed by EL Ghadoui et al. revealed that Inception V2 offered better generalization and superior accuracy and speed than ResNet-50, when employed for defect detection in injection-molded products [37].

Given these contradicting reports, it is challenging to select with certainty the optimal pre-trained CNN architecture for defect detection and classification in specular painted surfaces. For instance, ResNet has a unique non-sequential architecture with sizable depth that possesses lower complexity and training errors than other CNNs, since it employs skipping connections that substantially reduce the problem of vanishing gradients usually observed for deep CNNs [38]. Interestingly, while higher performance and accuracy would be expected in deeper ResNet networks such as ResNet-101, Park et al. revealed that a ResNet architecture with 50 layers was optimal, exhibiting higher accuracy when tested in several datasets of single-line and single-track metallic deposition of stainless-steel powders [39]. On the other hand, Inception V3 has an inception module that recognizes patterns of different sizes within the same layer, allowing it to detect a wide range of features in the input images and exhibit a remarkable performance in image classification tasks [40,41]. Furthermore, this architecture employs label smoothing, factorized 7 × 7 convolutions, and an auxiliary classifier to carry feature information throughout the network [42]. In this regard, in this work, a comparison will be performed between ResNet-50 and Inception V3 for defect detection and classification on the painted surfaces of heating devices. Furthermore, an additional comparison will be performed of these pre-trained models with a CNN built and trained from scratch to demonstrate the potential of TL for defect classification.

## 3. Proposed Methodology

### 3.1. Image Acquisition and Processing

#### 3.1.1. Experimental Setup

**Figure 1:**
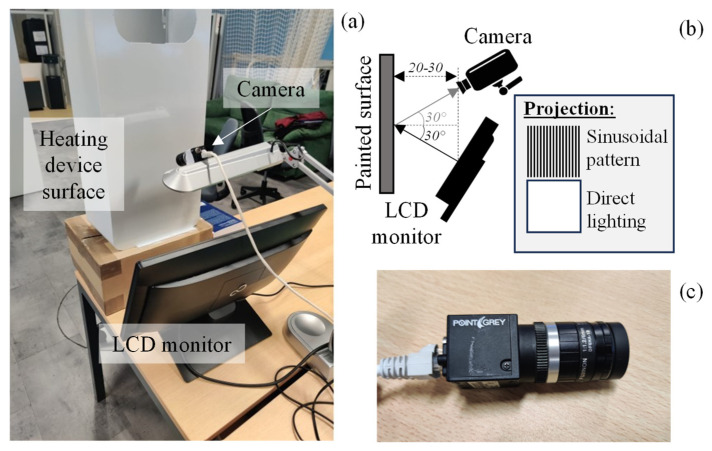
Experimental setup (**a**) and the respective schematic representation highlighting the two illumination modes used and the acquisition parameters (distance and angles) (**b**), and the camera employed (**c**). Distances are depicted in centimeters.

The experimental setup employed for image acquisition is depicted in Figure 1a and illustrated in Figure 1b. The liquid crystal display (LCD) monitor (21’’) provided two illumination modes: (1) structured illumination, by projecting a sinusoidal pattern; and (2) bright field illumination, by projecting a white screen. The camera then captured the imagens using both illumination modes in an alternate manner. The employed camera (BFLY-PGE-05S2C-CS, Point Grey Research Inc., Richmond, Canada), shown in Figure 1c, had a C-mount Fujinon lens with a fixed focal length of 6 mm for 1/2” sensors, that allowed for the capture of images with a resolution of 808 × 608. Image acquisition was powered via POE (Power over Ethernet) and performed using SpinView (Spinnaker SDK v. 1.27.0. 48). The selected configuration maintained the camera aligned with the LCD monitor at a distance between 20 (minimum) and 30 (maximum) centimeters from the painted surface, and at a 30° angle from the normal of the surface, resulting in a clearer projection with a more uniform lighting throughout the surface.

#### 3.1.2. Generation of the Sinusoidal Patterns

The sinusoidal patterns (Figure 1b) were generated in mathematical software Matlab, version R2021b, using the function (1), where *I* corresponds to captured image intensity (0 or 1), B to the ambient light intensity, A to the pattern’s amplitude, f to its frequency, and θ to its phase.

I(x) = B + A · cos(2πf x + θ)(1)

The frequency controlled the thickness of the strips, while the phase shifted the pattern in X or Y direction [4]. Two frequencies were used to generate patterns with different numbers of stripes: 0.8 Hz for 20 stripes, and 1.6 Hz for 40 stripes. The θ was 0 for both frequencies tested.

#### 3.1.3. Dataset Generation

Images of defective (NOK) and non-defective (OK) surfaces were captured with a resolution of 808 × 608, using the configuration detailed in Section 3.1.1. for both illumination modes. It should be emphasized that the image acquisition was performed on several occasions with significant variations in environmental lighting conditions attributed to daylight variations and weather (sunny and cloudy days) in order to replicate daylight variations visible in an indoor industrial complex. Furthermore, in order to scan the entire surface of the heating device, manual steering was necessary in order to capture images of all surfaces.

**Figure 2:**
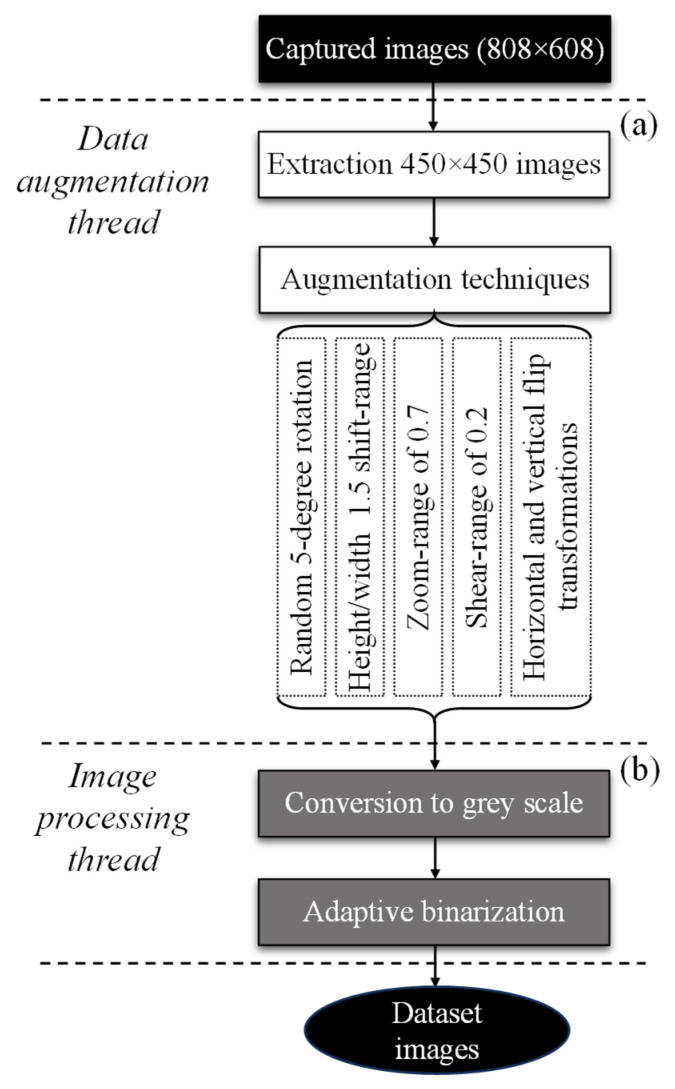
Data augmentation (**a**) and image processing (**b**) threads performed on the captured images.

The defect categories included scratches, dents, and spots with lack of paint. A detailed flowchart of the steps performed for the dataset generation is displayed in Figure 2.

Given the low availability of painted samples (with and without defects), two strategies were employed to augment the dataset to 500 images per defect category (Figure 2a): (1) four sub-images with 450 × 450 were extracted from the original 808 × 608 images using the GIMP software; and (2) several data augmentation techniques were then performed on the extracted sub-images, including a random 5-degree rotation, height and width shift-range of 1.5, zoom-range of 0.7, shear-range of 0.2, and horizontal and vertical flip transformations. Besides increasing the size of the dataset, these data augmentations also prevented data overfitting and improved the overall performance of the CNNs [10].

Afterwards, the images were subjected to processing through the conversion RGB to grey scale and the application of adaptive binarization to reduce the effect of the environmental lighting (Figure 2b). It should be emphasized that images obtained via bright field illumination were not subjected to processing.

From the total dataset generated, 80% of the images were used for training and the remaining 20% for testing. It should be emphasized that models were trained using images acquired with both illumination modes.

### 3.2. Feature Extraction and Classification Using Convolutional Neural Networks

All training and classification employed open sourced Keras framework with TensorFlow backend, implemented on a computer with an Intel^®^core i7 processor, a RAM memory of 8 GB, and a NVIDIA Geforce 930MX graphic card.

#### 3.2.1. Approach 1: Self-Built CNN

**Figure 3:**
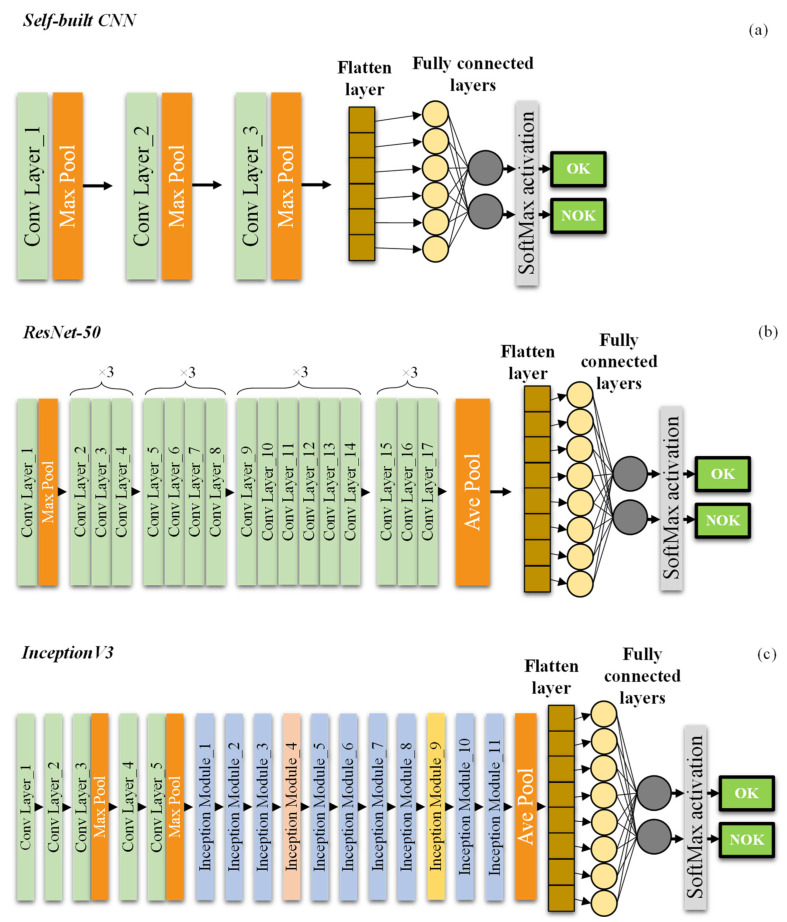
CNNs architectures employed: self-built (**a**), ResNet-50 (**b**), and Inception V3 (**c**).

The first approach consisted in the construction and training of a new and lightweight CNN from scratch. This network consisted of: (1) 3 CLs; (2) 3 max PL; (3) 1 flatten layer; and (4) 2 FCL. Every CL contained 32 filters with size of 3 × 3 and ReLu AFs. The first FCL consisted of 128 neurons and ReLu AFs, while the second FCL possessed 2 neurons and softmax AF to obtain two outputs of probability for OK or NOK classes, as depicted in Figure 3a. It should be noted that the CNNs’ default input image size was 450 × 450. This network was trained with an epoch value of 250.

#### 3.2.2. Approach 2: Pre-Trained CNNs

The second approach entailed the TL and fine-tunning of two common pre-trained CNNs: ResNet-50 and Inception V3. These networks have been trained on at least one million images from the ImageNet and have over 23 million trainable parameters [43]. The input image size of these pre-trained CNNs was also 450 × 450.

ResNet-50 is a residual network with 50 layers stacked on top of each other, comprising 48 CLs, 1 max PL, and 1 average PL. The last block of the network was fine-tuned with 1 FCL with 1024 neurons and 1 final FCL with only 2 neurons and softmax AF to obtain OK or NOK classes, as shown in Figure 3b. This network was trained with an epoch value of 300.

Inception V3 is a 48 layer-deep CNN architecture with inception modules that contain several convolutional and pooling layers in parallel to reduce the number of parameters. The last block of the network was fine-tuned with 1 global average PL, 1 FCL with 1024 neurons, and 1 final FCL with 2 neurons and softmax AF to obtain OK or NOK classes, as displayed in Figure 3c. This network was trained with an epoch value of 400.

### 3.3. Automation of the Defect Detection Process

**Figure 4:**
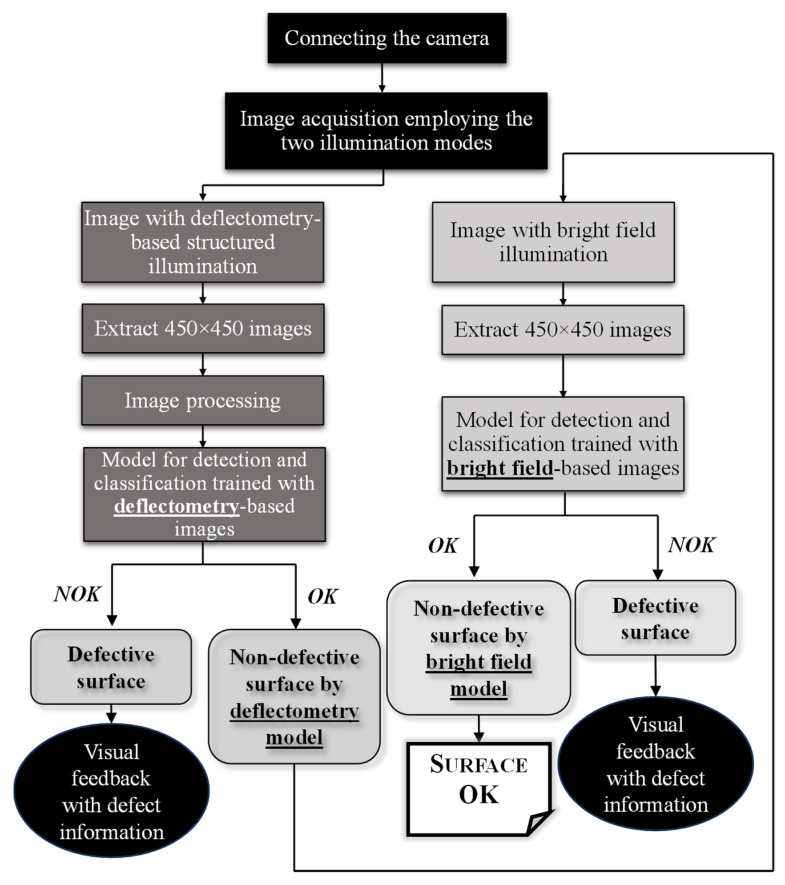
Flowchart detailing the steps of the automatic defect detection algorithm.

A Python-based algorithm was designed to automatize the entire defect detection and classification system, from the image acquisition to the predicted decision of the developed CNNs. The detailed flowchart is shown in Figure 4. Image acquisition and display were performed automatically in 5 s intervals employing both illumination modes. Smaller images with 450 × 450 pixels size were extracted from the images obtained with deflectometry-based structured illumination, and then processed to be input to the CNNs trained with the dataset of pattern-containing images. The detection module generated an output of OK or NOK surfaces, and the classification module identified the defect categories (scratches or dents). Once a surface was deemed OK using images with the sinusoidal pattern, a new algorithm cycle was performed employing images of the same surface illuminated with bright field. Since no processing was necessary for these images, images proceeded immediately from 450 × 450 extraction to the CNNs trained with dataset obtained with bright field illumination, in order to assess whether defects derived from lack of paint were detected.

It should be emphasized that this algorithm applied a decision-level fusion of the classification outputs, so for a painted surface to be classified as OK, its images should be submitted to CNNs trained with images of both illumination modes and obtain an OK classification on both. A confidence interval of 90% was required for the acceptance of a defect classification, given the overlap between the four subimages extracted from the original image.

**Figure 5:**
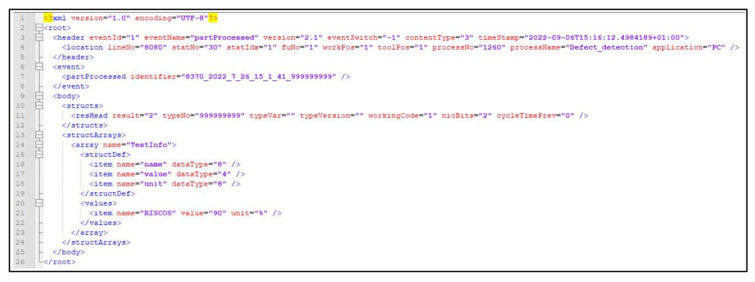
Telegram to be sent to MES.

Once the classification was performed, the information regarding the status and identification of the painted surface was sent via telegram to an existing manufacturing execution system (MES), implemented in the university, in order to simulate the industrial MES employed in an industrial sector. For a correct reception by MES, these telegrams exhibited a pre-defined general structure including: a header in the initial part of the message, where the name of the process (*Defect_detection*), eventName, timeStamp, location (specified the source of the information, such as production line and workstation) (Figure 5 on line 3 to 5); the element event, used to identify the part to which the information is related employing the 8370_YEAR_MONTH_DAY_HOUR_MIN_SEC_999999999 as the identifier nomenclature (Figure 5 on line 6 to 8); *resHead* structure, that contained the classification information, 1 if OK and 2 if NOK, followed the defect category employing nioBits in the case of NOK, defined as 2^code of defect category^ (a list with defect categories codes for nioBits match was previously imported to MES in a CSV file, identifying 1 as code to scratches, 2 to lack of paint, 3 to dents) (Figure 5 on line 11); and additional information about the defect, such as the defect designation and accuracy of the model’s classification (Figure 5 on line 13 to 24).

Afterwards, MES forwarded the telegram’s information to a graphical user interface (GUI). The GUI was also developed in Python, using a tkinter package, identifying the following parameters: identifier number of the painted surface, the image that was classified, the classification OK or the defect category, the total of already classified parts, date and time.

## 4. Results and Discussion

In this work, an automatic vision inspection system was developed for the detection and classification of defects on the painted surfaces of heating devices, performing image acquisition, processing, and decision-making regarding the status of those surfaces in an automated fashion. It should be emphasized that, despite being tested and optimized for the painted surfaces of heating devices and their different topographical features (specular and diffuse), this automatic system can be easily configured and optimized for any other painted surface, such as surfaces of cars, airplanes, or any other household appliance.

### 4.1. Dataset Acquisition and Generation

**Figure 6:**
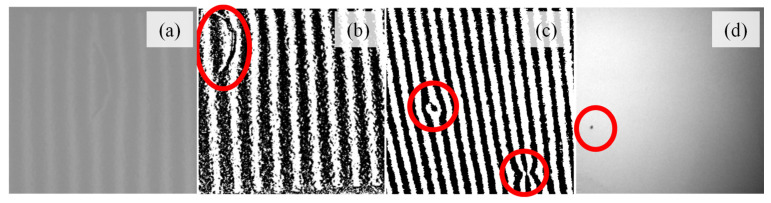
Image from the painted surfaces before (**a**) and after processing for scratches (**b**) and dents (**c**). Image from the painted surfaces with spots with lack of paint (**d**). The defects are highlighted by the red circles.

Environmental lighting was not controlled, in this experimental setup, to better approximate the lighting conditions observed in an industrial setting. In this instance, without proper containment of the acquisition setup, daylight variations could affect the light intensity in the captured images and alter the performance of the detection system. Indeed, as observed in Figure 6a, the low contrast observed in this image due to the interference of the environmental lighting hindered the immediate localization of the scratch. Nevertheless, the processing algorithm applied counteracted this effect, by generating high-contrast areas in the images containing sinusoidal patterns that, ultimately, facilitated the visualization of the defects, as seen in Figure 6b,c. Still, if containment is required for industrial implementation, tunnels such as those reported by Armesto et al., Molina et al., and Chang et al. could be considered [2,3,6].

The application of the two illumination modes—deflectometry-based structured illumination and bright field—was quite successful in the detection of all defects categories, despite their significantly different characteristics and sizes. Indeed, the deflectometry-based illumination was appropriate for the detection of defects with three-dimensional features such as scratches and dents in specular surfaces such as those obtained after the painting process, due to the distortion of the reflected pattern (Figure 6b,c) [2,4]. Conversely, defects derived from the lack of paint, which were much smaller (<0.5 mm), generate different superficial textures that were more easily detected using bright field (Figure 6d). In fact, using deflectometry-based illumination in this instance could trigger incorrect classifications, on the cases that the spots without paint appear in the black stripes of the sinusoidal patterns [18]. A similar multi-modal concept was previously reported by Guan et al. [7]. However, unlike Guan et al. that employed a feature-level fusion, a decision-level fusion method was applied in this work. This method not only preserved all the features extracted from the images retrieved using each illumination mode, but also significantly reduced the computational complexity of the final model by only fusing at the classification stage instead of fusing at the extraction stage [21]. Furthermore, this method is also compatible with real-time monitoring, which is essential for its implementation in an industrial inspection system, where time efficiency is required for increased industrial productivity.

Another important aspect of the image acquisition module is its ability to scan the entire surface of the heating device. In this work, however, a manual steering of the device was performed to expose all surfaces to the camera–monitor combo. Contemplating a totally automatic inspection process, a robotic arm, such as those reported by Akhtar et al. and Mao et al., should be considered [4,44].

### 4.2. Performance of the Defect Detection and Classification Models

**Figure 7:**
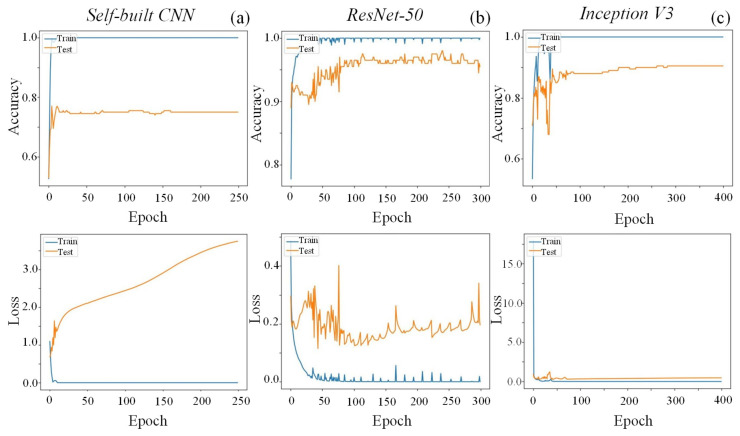
Accuracy (**upper**) and losses (**lower**) of the self-built CNN (**a**), ResNet-50 (**b**), and Inception V3 (**c**) during training and testing for scratch’s defect category, employing sinusoidal patterns with 20 stripes.

Firstly, the most efficient CNN for the detection and classification of defects in the painted surfaces was assessed for one of the defect categories—scratches, and the results are displayed in Figure 7. The self-built network achieved only 75% of accuracy and substantial loss throughout the 250 epochs (Figure 7a). In fact, when tested with images not used for training, this network prompted several incorrect predictions. Furthermore, this model exhibited a large gap between its training and testing losses’ curves, suggesting a possible overfitting problem. Similar results have been reported for models built from scratch [10,13], which can be attributed to the small dataset available for both training and testing in this work. As previously referred, given that defect occurrences are quite rare in most industrial sectors, the constitution of a sizable dataset is challenging, leading to problems of overfitting and unbalanced data [29,36,45]. TL and fine-tuning of pre-trained models were employed in this work to overcome this limitation. These pre-trained networks have shown higher prediction accuracy and possess more robust detection abilities than self-built CNNs [13,46], which was in fact confirmed in this work. Indeed, the fine-tuning of pre-trained CNNs considerably improved the outputs, with ResNet-50 and Inception V3 reaching around 95% and 90% of accuracy, respectively (Figure 7b,c). Furthermore, both models achieved their maximum accuracy values quite quickly within the iteration time (between 10 and 50 epochs), and they remained rather stable up to 300 epochs. These trends have already been reported for similar detection systems [10,11]. The superiority of pre-trained ResNet-50 for defect detection and classification has already been reported, and it has been attributed to its residual blocks, which significantly reduce the problems of vanishing gradients with increasing network’s depth, preventing overfitting and increasing the robustness of the model [47]. Hence, given its better performance, ResNet-50 was selected for defect detection and classification of the remaining defects.

**Figure 8:**
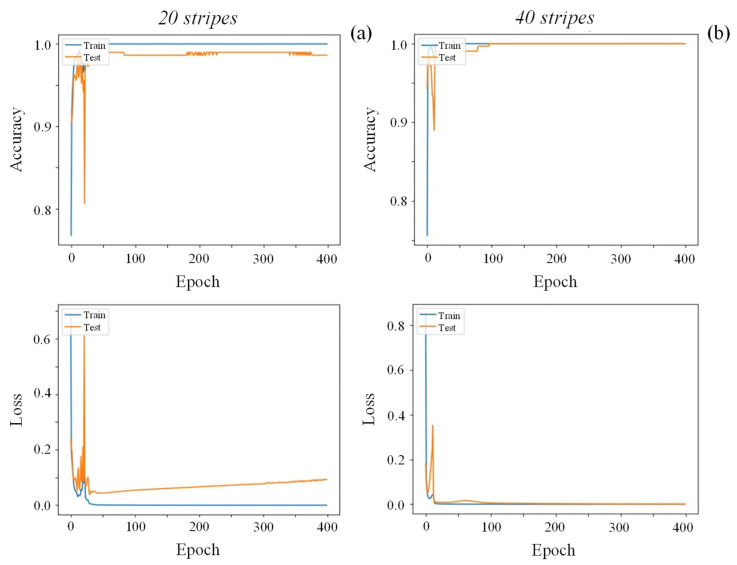
Accuracy (**upper**) and losses (**lower**) of ResNet-50 during training and testing using sinusoidal patterns with 20 (**a**) and 40 (**b**) stripes for the dent defect category.

Besides the type of CNN, the effect of different projected patterns on the detection and classification of the defects—in this particular case, dents—was also assessed by employing sinusoidal patterns with different stripe widths: 20 or 40 stripes in the same area. At this point, an improved model’s performance using thinner stripes was expected as it expanded the range of defect sizes that could be identified, as reported by Macher et al. [48]. This was confirmed by the evolution of the accuracy and losses of the model shown in Figure 8, that revealed a test’s accuracy improvement from 98 to 100% and considerable loss reduction and convergency by decreasing the stripes’ width.

**Figure 9:**
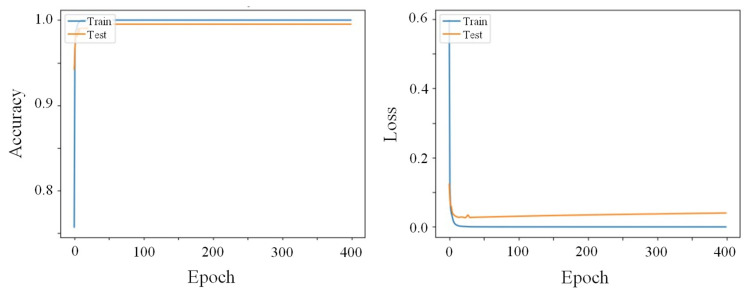
Accuracy and loss of the pre-trained ResNet-50 model during training and testing for the lack of paint defect category.

Regarding defects derived from lack of paint, whose results are shown in Figure 9, the model’s test performance reached 99% of accuracy with reduced loss after 400 epochs.

Overall, ResNet-50 performed well for the detection of all defect categories, with testing accuracies higher than 95%. Still, the network’s improvements should be performed, particularly regarding scratches detection and classification given its lowest accuracy and higher loss. In this instance, a reduction in the stripe width should be considered to enhance the model’s performance.

### 4.3. Performance of the Automatic Detection and Classification System

**Figure 10:**
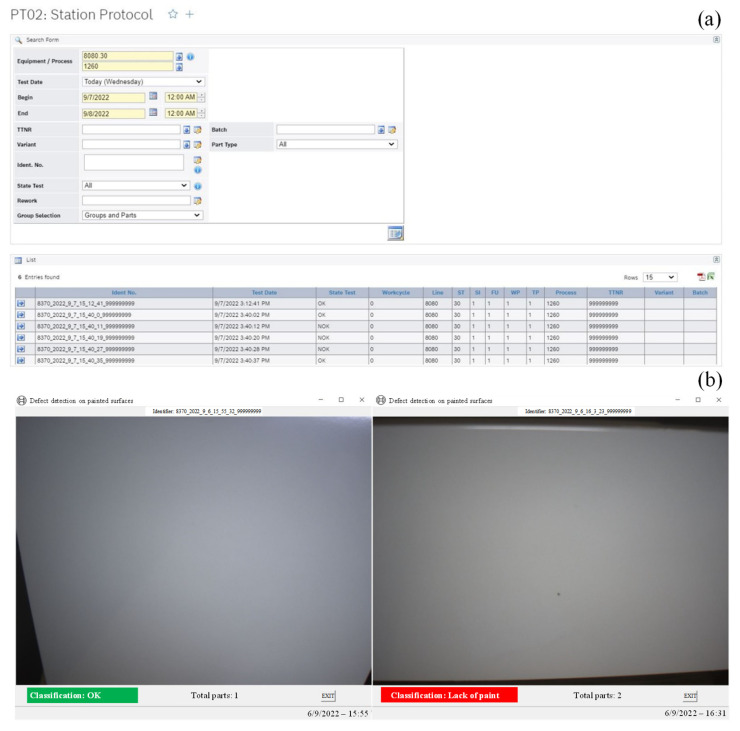
Telegram reception by the implemented MES (**a**), GUI for a surface OK and NOK due to lack of paint (**b**).

Given the real-time monitoring and classification required in an industrial inspection setting, the detection and classification system should be not only fast, but also accurate in its predictions [2,49]. In this work, once captured images were input into the models and the CNN was launched, defect classification was obtained in less than one second, which is suitable for an industrial implementation. Indeed, given their lower computational complexity, decision-level fusion models are quite adaptable for real-time applications and have a strong fault tolerance [21]. This defect information was then converted into a telegram and successfully sent to the MES server almost instantaneously without any errors. This was confirmed by reception of the telegrams on the implemented MES, exhibiting three OK and three NOK classifications, as shown in Figure 10a, indicating that three surfaces were defect-free while the following surfaces were NOK.

This information was then forwarded to the GUI. Figure 10b exhibit OK and NOK classifications of painted surfaces derived from light projection modes due to lack of paint. Similar visual interfaces have been developed for inspection systems [15,44]. In an industrial scenery, this GUI is highly significant, as it provides the information of painted surface status to the operators, that ultimately determines whether the product can continue in the production line or whether it needs to be removed and repainted or discarded.

All in all, the obtained results demonstrated the robustness and quickness of the developed inspection systems, from the image acquisition to the visualization through GUI, demonstrating its potential for implementation for industrial quality control of the painted surfaces. Despite all this, optimization of certain aspects of this system is still required to further improve its robustness. Concerning the dataset, it needs to be improved in terms of quality, with more captured images of defective and non-defective surfaces to accommodate each defect’s features’ real variations, and in terms of size, given that 500 images is still a small dataset for these models. Additionally, implementation of this system in an industrial painting line will be considered in the future.

## 5. Conclusions

An automatic inspection system was successfully developed for defect detection and classification on painted surfaces of heating devices, whose components exhibit different topographical features, requiring different illumination modes during acquisition. This system comprised deflectometry and bright light-based image acquisition for dataset generation, DL algorithms for defect detection and classification, surface’s information shipping to a MES server, and a final visual interface for defect information display. Three CNNs were tested: a lightweight CNN built and trained from scratch, and two fine-tuned pre-trained CNNs, ResNet-50, and Inception V3. From the experimental results, the following conclusions can be drawn:

1. The two illumination modes of the image acquisition module significantly widened the type of defects that could be identified with this system, while maintaining its computational complexity by performing multi-modal fusion at the decision level.
2. Pre-trained networks performed better than the self-built networks, with ResNet-50 exhibiting the best performance in terms of accuracy (higher than 95%) and speed for all defect categories.
3. Decreasing the sinusoidal pattern’s stripe’s width substantially improved ResNet-50’s accuracy and error convergency when applied for dented painted surfaces.
4. The entire sequence of the inspection system allowed for a fast (less than 1 s) and correct detection of all defect categories by imposing OK classification on models trained with images derived from both illumination modes: sinusoidal pattern and bright field.
5. The overall painted surface information was readily and correctly sent to the MES server, via telegram, which then forwarded it to a GUI.

All these conclusions validate the robustness and quickness of the system, proving that its implementation in the heating industrial sector would be an efficient tool for quality control of painted surfaces of heating devices.

## Data Availability

Datasets generated during the current study are available from the corresponding author on reasonable request.
